# The Effects of Forest Therapy on Coping with Chronic Widespread Pain: Physiological and Psychological Differences between Participants in a Forest Therapy Program and a Control Group

**DOI:** 10.3390/ijerph13030255

**Published:** 2016-02-24

**Authors:** Jin-Woo Han, Han Choi, Yo-Han Jeon, Chong-Hyeon Yoon, Jong-Min Woo, Won Kim

**Affiliations:** 1 Stress Research Institute, Inje University, Seoul 100-032, Korea; hanjw.stress@gmail.com (J.-W.H.); hanchoi.stress@gmail.com (H.C.); creativitylex@gmail.com (Y.-H.J.); jongmin.woo@gmail.com (J.-M.W.); 2 Department of Rheumatology, Uijeongbu St. Mary’s Hospital, Catholic University, Uijeongbu 480-717, Korea; chyoon@catholic.ac.kr; 3 Department of Psychiatry, Seoul Paik Hospital, Inje University School of Medicine, Seoul 100-032, Korea

**Keywords:** chronic widespread pain, forest therapy, autonomic nervous system, NK cell activity, depression, quality of life

## Abstract

This study aimed to investigate the effects of a two-day forest therapy program on individuals with chronic widespread pain. Sixty one employees of a public organization providing building and facilities management services within the Seoul Metropolitan area participated in the study. Participants were assigned to an experimental group (*n* = 33) who participated in a forest therapy program or a control group (*n* = 28) on a non-random basis. Pre- and post-measures of heart rate variability (HRV), Natural Killer cell (NK cell) activity, self-reported pain using the visual analog scale (VAS), depression level using the Beck Depression Inventory (BDI), and health-related quality of life measures using the EuroQol Visual Analog Scale (EQ-VAS) were collected in both groups. The results showed that participants in the forest therapy group, as compared to the control group, showed physiological improvement as indicated by a significant increase in some measures of HRV and an increase in immune competence as indicated by NK cell activity. Participants in the forest therapy group also reported significant decreases in pain and depression, and a significant improvement in health-related quality of life. These results support the hypothesis that forest therapy is an effective intervention to relieve pain and associated psychological and physiological symptoms in individuals with chronic widespread pain.

## 1. Introduction

In 1990, the American College of Rheumatology defined chronic widespread pain (CWP) as a condition in which pain is present for three months or more in at least five parts of the body. These parts comprise of the spine, and all quadrants that are defined by an axis through the waist and an axis through the spine [1]. The etiology of CWP is unknown. Besides the experiencing of musculoskeletal pain felt throughout the body, this condition is often associated with both physical and psychological problems including anxiety, depression, fatigue, sleep problems, and lower quality of life [2,3]. Internationally, the prevalence of CWP is reported to be 10%–14% of the world’s population, and is known to be more common in women than in men [4].

Pain, which is an unpleasant sensation or emotional experience, has not received as much attention from researchers as other symptoms. This is because pain is a subjective experience, so it is difficult to quantify, and it is commonly thought that pain does not directly threaten human life [5,6]. However, pain causes inconvenience in daily life and thus can interfere with economic and social activities. As more and more emphasis and value has been put on quality of life recently, the treatment and management of pain has been receiving increased attention as well [7,8,9,10].

In Korea, the number of patients experiencing CWP and the costs associated with this condition is increasing each year [11,12]. In the case of the United Kingdom, it has been reported that medical expenses spent on CWP are similar to expenses spent on major diseases including cancer, cardiovascular diseases, and diabetes. It is estimated that associated costs due to the loss of work productivity amount to tens of billions of pounds [13]. Thus, CWP has become an ailment that can no longer be neglected.

Until now, commonly used pain treatment methods have included pharmotherapy, exercise, psychological treatment, surgery, and complementary and alternative medicine [14,15,16]. Among these methods, psychological treatment has been most commonly used to effectively treat CWP. According to past studies on CWP, psychologically-focused multidisciplinary treatment is known to be effective in alleviating negative emotions that are associated with pain [2,13,15]. One of the reasons why a psychologically-focused approach is effective is that the cause of CWP is etiologically unclear, and the physical and psychological deterioration resulting from experiencing pain for three months or more requires treatment approaches from diverse perspectives. Additionally, as already mentioned, pain is associated with unpleasant sensations or negative emotional experiences, and thus deeply related to human emotions. As pain is a subjective phenomenon by nature, psychological approaches can be relevant [17]. Results from the past studies show that psychological interventions such as cognitive behavioral treatment have an important role in the treatment of CWP.

Psychological interventions seem to be effective in diminishing pain and comorbid psychological problems of CWP, because they induce changes in the perception of pain [14,15,17,18]. A physical activity component would be a useful addition to standard treatments for an individual to gain a sense of control about pain and to reduce tension from nervous system which can co-occur with the exacerbation of pain [13,14].

Forest therapy which utilizes psychological approaches and appropriate physical activities in a natural environment to improve pain and comorbid psychological complications may be a useful resource for treatment of CWP [16,19]. Currently, an increasing number of studies are providing evidence for the effectiveness of forest therapy in various psychological and physical symptoms. Forest therapy has been shown to be effective in providing physical relaxation, soothing anxiety, and relieving depression symptoms as well as activating the parasympathetic nervous system. It has also been shown to have positive effects on neurocognitive functioning and emotion [20,21,22]. With continued publications of such studies, forest therapy has gained increasing recognition as an intervention method based on scientific evidence [23,24,25].

In the current research, we evaluated the effectiveness of a forest therapy program that was specifically designed for patients with CWP. The overall aim of the program is to provide psycho-education regarding pain management and to enhance motivation for long-term lifestyle changes. The two-day program consists of educational and various guided physical activities in the forest based on cognitive behavioral therapy, mindfulness-based meditation, savoring natural olfactory and auditory stimuli, relaxation, and music therapy.

Forest therapy consists of various factors including a psychological approach, physical activity, and the provision of a safe and restorative environment in which an individual can independently experience cognitive change. We hypothesized that, forest therapy is an effective treatment method for CWP, a condition that requires a multidisciplinary treatment approach.

## 2. Materials and Methods

### 2.1. Participants and Design

Study participants were full-time employees (age 25–49) from a public organization that provided management for buildings and facilities in the Seoul Metropolitan area who were experiencing CWP. Approval for the study was obtained from the Inje University Seoul Paik Hospital Institutional Review Board (IRB: IIT-2014-168) prior to initiating the study. Three trials were conducted for this study. Each trial was conducted with a different group of participants, with each participant only taking part in one of three trials. An initial survey with questions about pain level was administered to the employees to recruit participants. Moreover, willingness to participate in the forest therapy was obtained from an initial survey. Inclusion criteria for this study were: pain is present for three months or more in at least five tender points, full-time employee with no history of psychiatric disorder, female participants who had not yet begun menopausal transition, and no history with severe allergic reaction exposed by forest environment.

For the first trial, 268 employees were surveyed from 2 May 2014 to 9 June 2014. For the second trial, 283 employees were surveyed from 21 August 2014 to 5 September 2014. For the third trial, 127 employees were surveyed from 14 July 2015 to 28 August 2015. During recruitment, participants were clearly informed about the study purpose and procedure.

A rheumatologist was then consulted to identify eligible candidates who met the inclusion criteria and were willing to participant in forest therapy for the study purpose. Those who agreed to participate were then selected as study participants who gave full-written consent for taking part in the first (experimental group *n* = 11, control group *n* = 11), second (experimental group *n* = 13, control group *n* = 8) trial, and third (experimental group *n* = 9, control group *n* = 9) experiments. 

As all the participants are working in a same company of whom the occupational roles are similar, these two groups seemed to share many characteristics in common. While recruiting the control group, we tried to achieve a 1:1 match in terms of age and gender distribution.

An overview of the demographic data as well as duration of suffering pain is shown in Table 1. Overall, the experimental and control groups were quite comparable, and there were no significant differences except age and hours of sleep in the demographic data.

This study did not account for the possible impact of menstrual cycle, which may influence participating female in terms of immunity, chronic pain and autonomic activity.

### 2.2. Experimental Treatment

**Figure 1:**
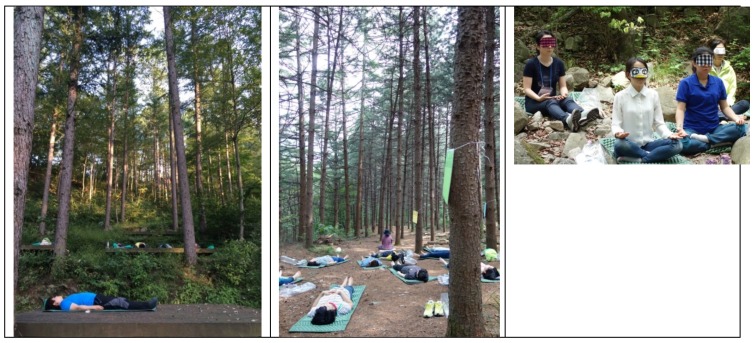
Impressions of therepeutic activities in the forest environment. Photographs courtesy of Haejung Kim.

The three forest therapy camps were held at the Saneum Natural Recreation Forest in Yangpyeong county of Gyeonggi Province. Saneum Natural Recreation forest is located at the base of Danwol-myeon Saneum-ri Bongmisan Mountain. This forest is very lush and the valley boasts spectacular views. The list of trees includes pine, oak, and maple (See Figure 1). The weather on the days of this study was sunny, and the average temperatures were 20.35 ± 2.4 °C in the forest environment, 22.2 ± 1.2 °C in the urban environment. The average humidity was 61.1% ± 18.9%, the average speed of wind was 1.01 ± 0.26 m/s and the average sunshine duration was 6.33 ± 2.4 h per day in the forest environment, whereas in the urban environment, the average humidity was 61.1% ± 18.9%, the average speed of wind was 2.05 ± 0.45 m/s and the average sunshine duration was 6.97 ± 4.14 h per day. 

Each forest therapy camp lasted two days. During those two days, the participants engaged in various indoor and outdoor activities aimed at providing relaxation, refreshment, and attention restoration. The activities were supervised by a professional team consisting of one psychiatrist, one rheumatologist, one forest guide, and one forest therapist.

### 2.3. Physiological Indices

Physiological assessments included cardiac measures and natural killer (NK) cell activity. Heart rate and its variability (HRV) is a physiological marker that provides an outlook on sympathetic and parasympathetic nervous system functioning [26]. As for the sympathetic activation, the measures of HRV are accompanied by increase in Heart Rate (HR) and decrease standard deviation of normal to normal intervals (SDNN) and Total Power (TP). However in return, activation of parasympathetic activity reflects decrease in HR and increase in SDNN and TP. Participants were fitted with a long-term electrocardiogram (ECG) R-R interval (RRI) T-REX^®^ (Monitor and Care; Taewoong Medical, Gyeonggi do, Korea) recorder and a customized electrocardiography electrode to measure HRV continuously during the entire experimental period. Resting coherence ratio measurement was used to collect 10 min of heart-rhythm data after 10 min resting periods in a sitting position to monitor participants’ pre- and post- results. Participants were restricted from consuming coffee, tea, or other caffeinated beverages during one-hour prior to the baseline and end point measurements. Cigarettes were also restricted for a minimum of 30 min before the recording. General measures including heart rate and specific measures of HRV such as standard deviation of normal to normal intervals (SDNN) and total power (TP) were analyzed to assess autonomic arousal and ANS function [27,28].

Cytotoxic activity of NK cells was determined using the NK Vue-Kit^®^ (ATgen, Sungnam, Korea). NK cell activity is an immunological marker that effected by various psychological conditions [29]. 10 mL of whole blood was collected using BD Vacutainer^®^ heparin N1 tubes. 1 mL of whole blood was incubated for 24 h, at 37 °C, under 5% carbon dioxide (CO_2_) with indicated dose of Promoca^®^ and 1 mL of RPMI 1640 media. Cell-free supernatants were harvested, and NK cell activity levels were determined according to manufacturer’s protocols [30].

### 2.4. Psychological Indices

Self-reports of pain and other psychological indices were obtained via a written questionnaire, which was administered during the pre- and post-measurements. Pain was measured on a Visual Analog Scale for Pain (VAS Pain), with scores ranging from 0 = least possible pain to 10 = worst possible pain [31]. Participants also filled out the Beck Depression Inventory (BDI), with scores ranging from 0–13 = minimal depression, 14–19 = mild depression, 20–28 = moderate depression to 29–62 = severe depression [32,33], and the EuroQol Visual Analog Scale (EQ-VAS) [34] a measure of health related quality of life, with scores ranging from 0 = worst imaginable health state to 100 = best imaginable health state. The questionnaire also included questions on demographic variables like gender, age, and education level.

### 2.5. Procedure

The first camp was held from 19 to 20 June in 2014, the second from 18 to 19 September in 2014 and the third from 21 to 22 September in 2015. The first day started with pre-test measurements at Inje University Seoul Paik Hospital in the urban environment. After the HRV recorder was fitted, the participants filled out questionnaires with psychological indices, and blood samples were collected for NK cell activity. After a two-hour drive to the forest and a lunch, the program started with walking and therapeutic activities in the forest. The evening program of the first day consisted of indoor music therapy and a psychoeducation on coping with pain and stress. Participants stayed overnight at the lodge in the Saneum Natural Recreation Forest. The morning program of the second day consisted of bodily exercises and mindfulness-based meditation in the forest. After lunch, the program ended with post-test measurements with identical sequences to the pre-test in the Saneum Natural Recreation forest auditorium. See Table 2 for a detailed overview of the forest therapy program. Participants in the control group were asked to come to Inje University Seoul Paik Hospital twice on two consecutive days on the weekend to engage in an identical set of pre- and post-measurements. At the time of the study, the control group was not engaged in any psychological or therapeutic treatments for CWP. The control group was instructed to perform usual weekend routines except visiting natural environments such as urban parks or forest environments. Moreover the control group was instructed not to conduct either heavy loads of domestic or occupational work during the enrollment in this study. The data were obtained from the control group no further than three weeks away from the experimental sessions.

### 2.6. Statistical Analyses

All statistical analyses were performed using SPSS 21.0 (IBM corporation, Armonk, NY, USA). First of all, chi-square test and the independent *t*-test were used to test for possible differences between two groups at baseline. Differences between the experimental and control groups were analyzed with Repeated Measures ANOVA with time of measurement (pre, post) as a within factor and group (experimental, control) as a between-subjects factor. Post-hoc analyses of differences between pre- and post-measurements within groups were conducted with paired sample *t*-tests. In case of baseline differences, additional analysis of covariance (ANCOVA) were conducted on the post-test measures with group (experimental, control) as a between-subjects factor and the baseline values as covariates.

## 3. Results

### 3.1. Physiological Measures

Table 3 provides an overview of the mean scores on all physiological measures in the two groups at the two times of measurements. A series of *t*-tests revealed that there were significant baseline differences in HR and NK cell activity between the experimental and the control group, in such a way that HR and NK cell activity were higher in the control than in the experimental group, *p* < 0.05. For the other physiological measures (SDNN, TP) there were no significant differences between the experimental and control groups at baseline.

**Figure 2:**
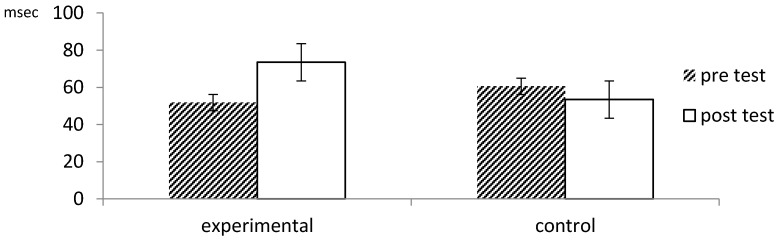
Effect on forest therapy on SDNN.

Repeated measures analysis of the SDNN data showed a significant main effects of time, *F* = 7.10, *p* = 0.01, *η_p_*^2^ = 0.11. This main effect was however qualified by a significant interaction effect between time and group on SDNN, *F* = 28.16, *p* = 0.000, *η_p_*^2^ = 0.34. As illustrated in Figure 2, there was a strong and significant increase in SDNN within the experimental group, *p* = 0.000, favoring a relaxation response and improvement of ANS function, while there was a small but significant decrease in SDNN in the control group, *p* = 0.014.

**Figure 3:**
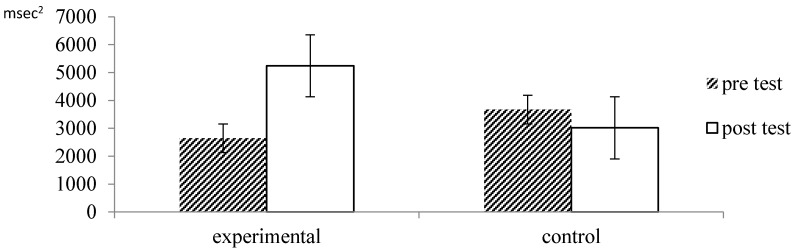
Effect of forest therapy on TP.

The TP data also showed a significant main effects of time, *F* = 5.90, *p* = 0.018, *η_p_*^2^ = 0.10, which was qualified by an interaction by time and group, *F* = 16.43, *p* = 0.000, *η_p_*^2^ = 0.23. As illustrated in Figure 3, there was a strong and significant increase in TP within the experimental group, *p* = 0.000, while there was a small decrease in TP in the control group which was not significant, *p* = 0.097. Thus, consistent with our hypotheses, both measures of HRV (SDNN and TP) suggest that participation in forest therapy leads to significant increases of heart rate variability and enhanced cardiac autonomic activity, which suggests a relaxation response of participants.

**Figure 4:**
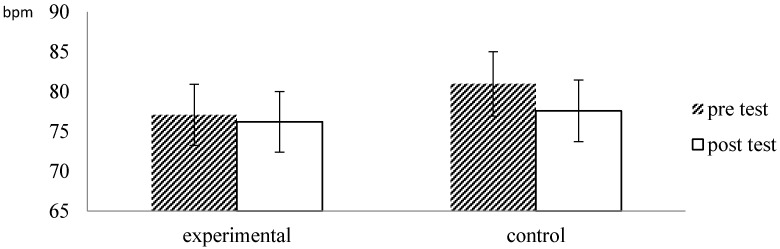
Effects of forest therapy on HR.

HR data showed main effects of time, *F* = 7.88, *p* = 0.007, *η_p_*^2^ = 0.12, and group, *F* = 2.44, *p* = 0.12, *η_p_*^2^ = 0.04, indicating that, on average, HR decreased from pre- to post measurement, and was higher in the control group than in the experimental group. As illustrated in Figure 4, there was a marginally significant interaction effect between time and group on HR, *F* = 2.73, *p* = 0.10, *η_p_*^2^ = 0.05, indicating that HR was significantly decreased within the control group, *p =* 0.021, while it did not change in the experimental group, *p* = 0.279.

**Figure 5:**
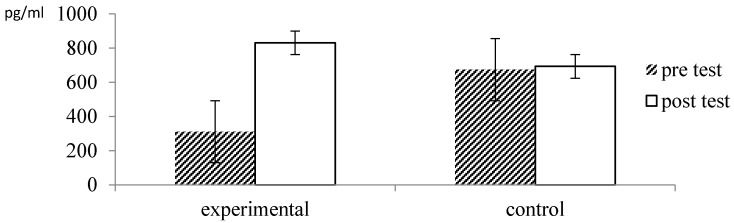
Effect of forest therapy on NK cell activity.

NK cell activity generally increased from pre- to post measurement, as indicated by a significant main effect of time *F* = 26.83, *p* = 0.000, *η_p_*^2^ = 0.31. This increase in NK cell activity differed significantly between the two groups, as indicated by a significant interaction between time and group, *F* = 9.99, *p* = 0.002, *η_p_*^2^ = 0.15. As illustrated in Figure 5, participants in the forest therapy group showed a significantly bigger increase in NK cell activity than participants in the control group, which was consistent with the hypotheses.

### 3.2. Psychological Measures

Table 4 provides an overview of the mean scores on all psychological measures in the two groups at the two times of measurements. A series of t-tests revealed that there were no significant baseline differences in self-reported pain (VAS Pain), depression (BDI), and health-related quality of life (EQ-VAS) between the experimental and the control group, *p* > 0.16.

**Figure 6:**
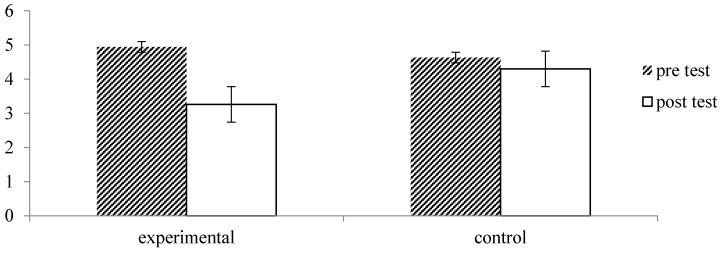
Effect of forest therapy on the Visual Analog Scale for Pain (VAS Pain), with scores ranging from 0 = least possible pain to 10 = worst possible pain.

Repeated measures analysis of the VAS Pain data showed a main effect of time, *F* = 29.27, *p* = 0.000, *η_p_*^2^ = 0.33, which was qualified by a significant interaction with group, *F* = 13.50, *p* = 0.001, *η_p_*^2^ = 0.19. As illustrated in Figure 6, participants of the forest therapy program reported a significant decrease in pain from pre- to post measurement, *p* = 0.000, while this decrease was small and non-significant in the control group, *p* = 0.25.

**Figure 7:**
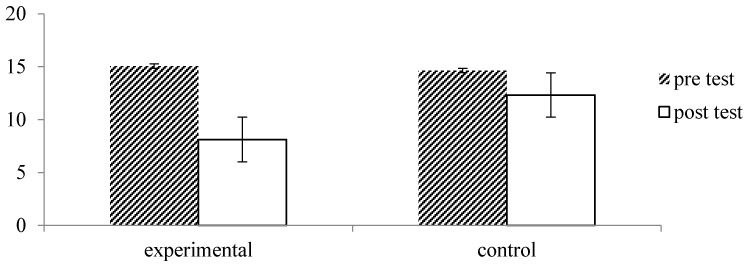
Effect of forest therapy on depression, measured by BDI, with scores ranging from 0–13 = minimal depression, 14–19 = mild depression, 20–28 = moderate depression to 29–62 = severe depression.

Also, repeated measures analysis of the BDI data showed a main effect of time, *F* = 45.62, *p* = 0.000, *η_p_*^2^ = 0.44, which was qualified by a significant interaction with group, *F* = 11.34, *p* = 0.001, *η_p_*^2^ = 0.16. As illustrated in Figure 7, participants of the forest therapy program reported a significant decrease in depression from pre- to post measurement, *p* = 0.000, while this decrease was significant in the control group, *p* = 0.015.

**Figure 8:**
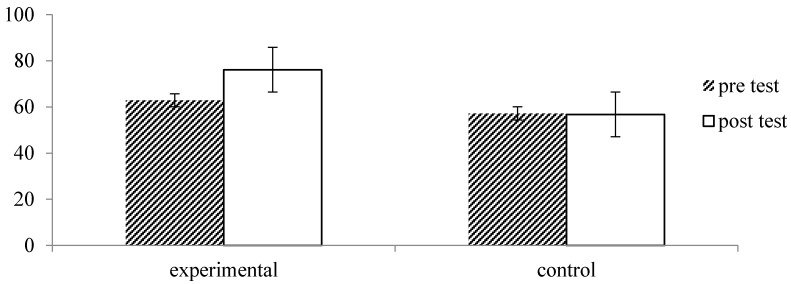
Effect of forest therapy on health-related quality of life, measured by EQ-VAS, with scores ranging from 0 = worst imaginable health state to 100 = best imaginable health state.

Regarding health-related quality of life, measured by EQ-VAS, there were significant main effects of time, *F* = 13.82, *p* = 0.000, *η_p_*^2^ = 0.19, and group *F* = 6.53, *p* = 0.013, *η_p_*^2^ = 0.1, which were qualified by an interaction by time and group, *F* = 15.91, *p* = 0.000, *η_p_*^2^ = 0.21. As illustrated in Figure 8, there was a significant increase in EQ-VAS within the experimental group, *p* = 0.000, while EQ-VAS did not change in the control group, *p* = 0.88. Thus, consistent with our hypotheses, participation in forest therapy can lead more beneficial effects to human health in reducing subjective pain and depression, improving health-related quality of life, and enhancing ANS function and immunity than doing nothing.

HR data showed main effects of time, *F* = 7.88, *p* = 0.007, *η_p_*^2^ = 0.12, and group, *F* = 2.44, *p* = 0.12, *η_p_*^2^ = 0.04, indicating that, on average, HR decreased from pre- to post measurement, and was higher in the control group than in the experimental group. As illustrated in Figure 4, there was a marginally significant interaction effect between time and group on HR, *F* = 2.73, *p* = 0.10, *η_p_*^2^ = 0.05, indicating that HR was significantly decreased within the control group, *p* = 0.021, while it did not change in the experimental group, *p* = 0.279. Additional ANCOVA analyses revealed that there were no differences at post-test between the experimental and control groups while controlling for baseline differences, *F* = 0.59, *p* = 0.45, *η_p_*^2^ = 0.01.

NK cell activity generally increased from pre- to post measurement, as indicated by a significant main effect of time *F* = 26.83, *p* = 0.000, *η_p_*^2^ = 0.31. This increase in NK cell activity differed significantly between the two groups, as indicated by a significant interaction between time and group, *F* = 9.99, *p* = 0.002, *η_p_*^2^ = 0.15. As illustrated in Figure 5, participants in the forest therapy group showed a significantly bigger increase in NK cell activity than participants in the controlgroup, which was consistent with the hypotheses. Additional ANCOVA analyses revealed that, controlling for baseline differences, there was significantly more NK cell activity at the post-test in the experimental than in the control group, *F* = 10.06, *p* = 0.002, *η_p_*^2^ = 0.15.

## 4. Discussion

This study aims to understand the physiological and psychological effects of forest therapy on adults who suffer from CWP, a pain condition with a range of disabling symptoms and substantial negative social and economic impacts. Three separate occasions of forest therapy camps were conducted to relieve pain and resolve secondary physiological symptoms and psychological problems (*i.e*., depression and decreased health-related quality of life) related to pain. Pre- and post- values for a control group were measured for comparison.

Consistent with the hypotheses, participants of the forest therapy program, as compared to a control group, showed more physiological relaxation as indicated by a bigger increase in HRV (as measured by SDNN and TP) as well as an enhanced immune competence as indicated by a stronger increase in NK cell activity. Participants in the forest therapy group also reported stronger decreases in pain and depression, and stronger increases in health-related quality of life, than participants in the control group. Thus, the findings of the present research add to the evidence-base for health benefits of forest therapy [35,36,37] and confirm its relevance and clinical importance for patients with CWP, a patient group that has thus far received no attention from research on forest therapy.

The results from the present research do not provide insights into the components of forest therapy that contribute to its positive effects on CWP. Previous research suggests that therapeutic elements of forest environments including sound of gentle birds and water stream, smell of phytoncides (various bactericidal substances obtained from plants) of a forest, scenery of colorful nature, touch of fresh air, and taste of forest products are basic contributors to restorative and invigorating effects of forest therapy [38,39]. In addition, forest therapy provides opportunities to avoid conflicts from strong internal and external stimuli in urban environments and ensures a safe space other than the stimuli in various forest environments to awaken the senses [38]. It is also likely that the activities that were offered in forest therapy program, such as mindfulness-based mediation, walking, and other activities promoted more stable changes in ANS functioning, anxiety, anger, fatigue, and depression, and thereby added to the effects of the natural environment [20,21,24,35,40].

Moreover, there were clear differences between the forest therapy group and the control group. Therefore, it can be suggested that a forest environment and forest therapy program improve the health status and health-related quality of life among CWP patients. This finding is consistent with the results of studies showing that frequent users of a forest environment showed higher ANS function and psychological restoration than a comparison group using it at a lower frequency [21]. 

In sum, there is no precedent research of forest therapy on CWP. However, in the application of mindfulness based meditation on chronic back pain [41], the medium size of effect was found for health-related quality of life, depression and pain perception, whereas the large size of effect was found for health-related quality of life in this study. Thus, the effect sizes found in this study are quite compatible to the effects sizes reported in other studies on comparable pain-therapies.

However, we fully acknowledge the limitations of this study. The first limitation is that it may not be possible to generalize the results to all patients with CWP. As the participants were recruited from workers in a public service organization, most of the participants showed moderate levels of pain symptoms. More severe patients who cannot maintain a full-time job were not included in this study. As some patients with CWP tend to suffer from a chronic course and notoriously widespread multiple tender points, the period of one over-night therapy weekend might be insufficient for more severe patients with CWP. Careful consideration should be given to the implementation of forest therapy for people with severe symptoms who have difficulty in social and family life due to CWP.

Secondly, we could not measure the long-term effects of the forest therapy program. Further research on the longitudinal effect of forest therapy after returning to daily routine and the need of booster sessions needs to be conducted via a follow-up study. In addition, the effects of exposure to the forest environment and the therapeutic activities could not be distinguished in this study. By including more active control groups that receive similar therapy in a non-natural environment future research might be able to gain more insight into the contribution of the forest environment to the beneficial effects of forest therapy. 

Thirdly, there were some limitations in the process of random assignment which may have weakened the internal validity of the study, including potential non-equivalence of groups at pre-test (*i.e*., on variables not assessed).

Fourthly, we did not measure individual effects and weights of each therapeutic treatments included in the forest therapy program. We hope that this study may open up further studies of comparing individual therapeutic treatments performed in the forest environment.

Lastly, the control group in the study was a waiting list control and did not engage in any type of therapy; therefore, the study does not speak to whether forest therapy is better than other therapies. We also cannot exclude the possibility that the result of this study might be partly due to a placebo effect resulting from positive expectations and beliefs about the positive effects of the forest therapy.

## 5. Conclusions

The results indicate that forest therapy can help to improve psychological and physiological symptoms of chronic widespread pain for which clear therapeutic methods have yet to be provided. This study may thereby contribute to promoting individual health and forming a healthy culture by proposing forest therapy as a means of managing and improving pain and physical and mental stability with pleasant individual activities.

## Figures and Tables

**Table 1:** Demographic characteristics and pain measurements in an experimental and control group.

Parameter		Mean (Standard Deviation)		*p*
		Experimental Group	Control Group	
Total sample number		33	28	
Sex	Male	16	10	0.315
	Female	17	18	
Age (years)		41.6 (6.5)	37.5 (8.4)	0.035 *
Height (cm)		166.8 (8.2)	165.4 (8.0)	0.507
Weight (kg)		66.7 (14.6)	63.21 (13.5)	0.341
Smoking	Yes/No	10/23	5/23	0.232
Drinking	Yes/No	21/10	20/8	0.759
Working Type	Day work	27	25	0.443
	Two shifts	3	3	
	other	3	0	
Working hours		8.3 (0.7)	8.6 (1.0)	0.167
Economic status	Fair	21	19	0.730
	poor	12	9	
Sleep duration (hours per day)		5.9 (1.1)	6.5 (0.9)	0.038 *
Duration of suffering pain (month)	3 months or less	3	7	0.504
	3–6 months	4	3	
	6–12 months	9	5	
	12–24 months	4	2	
	24 months or more	13	11	

Note: * *p* < 0.05.

**Table 2:** Forest therapy camp program.

Time	Program	Location
Day 1		
09:00–11:00	Orientation and Pre-test	Seoul Paik Hospital (Indoors)
11:00–13:00	Going to forest	
13:00–14:00	Lunch	Saneum Natural Recreation Forest (Outdoors)
14:00–16:00	Walk in the forest and forest activities	
16:00–17:30	Free time	
17:30–19:00	Dinner	
19:00–20:00	Music therapy	Saneum Natural Recreation Forest Auditorium (Indoors)
20:00–21:00	Psychoeducation: Coping with pain and stress	
Day 2		Saneum Natural Recreation Forest (Outdoors)
08:30–09:30	Breakfast	
09:30–11:00	Stimulation bodily exercise	
11:00–11:30	Mindfulness-based meditation	
11:30–12:30	Herbal tea time	
12:30–13:30	Lunch	
13:30–15:00	Post-test	Saneum Natural Recreation Forest Auditorium (Indoors)

**Table 3:** Comparison of physiological variables pre-post test between experimental and control group.

Variable	Sub-Factor	Group	Mean (Standard Deviation)			
			Pre Test	Post Test	*t*	*p*
ECG	**SDNN**	Experimental (*n* = 32)	51.86 (19.55)	73.50 (29.17)	−4.959	0.000 ***
		Control (*n* = 26)	60.60 (21.37)	53.43 (19.90)	2.643	0.014 *
	**TP**	Experimental (*n* = 32)	2645.43 (1898.77)	5244.58 (4185.12)	−3.977	0.000 ***
		Control (*n* = 26)	80.98 (8.06)	77.59 (7.55)	2.467	0.021 *
	**HR**	Experimental (*n* = 32)	77.09 (6.30)	76.21 (6.23)	1.102	0.279
		Control (*n* = 26)	80.98 (8.06)	77.59 (7.55)	2.467	0.021 *
**NK cell**		Experimental (*n* = 33)	604.20 (754.92)	1131.56 (990.29)	−5.391	0.000 ***
		Control (*n* = 28)	1067.16 (908.15)	1194.80 (996.99)	−1.715	0.098

Note: ECG: electrocardiogram, SDNN: standard deviation of normal to normal intervals, TP: Total Power, HR: Heart Rate, NK cell: Natural Killer cell, * *p* < 0.05, *** *p* < 0.001.

**Table 4:** Comparison of psychological variables pre/post test between experimental and control group.

Variable	Group	Mean (Standard Deviation)			
		Pre Test	Post Test	*t*	*p*
VAS Pain	Experimental (*n* = 33)	4.94 (1.62)	3.26 (1.69)	6.681	0.000 ***
	Control (*n* = 28)	4.63 (1.92)	4.30 (2.10)	1.185	0.246
BDI	Experimental (*n* = 33)	15.06 9.43)	8.12 (7.05)	6.869	0.000 ***
	Control (*n* = 28)	14.64 (9.67)	12.32 (9.99)	2.601	0.015 *
EQ-VAS	Experimental (*n* = 33)	62.88 (16.78)	76.09 (16.34)	−7.798	0.000 ***
	Control (*n* = 28)	57.21 (23.14)	56.75 (24.35)	0.148	0.884

Note: BDI: beck depression inventory, * *p* < 0.05, *** *p* < 0.001.
